# The Elements of Materia Medica and Therapeutics

**Published:** 1852-01

**Authors:** 


ART. XIV.—*The Elements of Materia Medica and Therapeutics.* By JONATHAN PEREIRA, M, D., F. R. S. and L. S. Third American edition, enlarged and improved by the author. Including notices of the mechanical substances in use in the civilized world, and forming an Encyclopedia of Materia Medica. Edited by Joseph Carson, M. D., Professor of Materia Medica and Pharmacy in the University of Pennsylvania. Vol. I. Philadelphia: Blanchard & Lea. 1852.

Dr. Pereira has performed an acceptable service in revising his great work on Materia Medica, a work that has no rival in the English language. The improvements that are continually adding to the resources of Materia Medica, and Therapeutics, render new editions, even of such master works as this, occasionally necessary. Dr. Pereira says: "Messrs. Blanchard & Lea, the respectable publishers of Philadelpnia, having informed me of their intention to bring out a new edition of my Elements of Materia Medica, for the American market, I undertook the correction of the last London edition, the first volnme of which was published in 1849."

"The recent publication of new editions of the London and Dublin Pharmacopœias rendered necessary a careful revision of the formulæ of the British Colleges, and entailed on me no trifling labor."

"I have embodied all recent discoveries of importance which relate to the subjects treated of in this work."

The editor, Professor Carson adds: that "he has performed the duty of introducing the improvements and emendations made in the recently issued edition of the United States Pharmacopœia, as well as notices of articles, and information upon topics, which more especially interest American physicians."

The author and editor are probably as well qualified as any men living to get up a standard work on Materia Medica and Therapeutics, and we commend this work as an invaluable accession to any medical library. It is complete in all its departments, and is well entitled to the study of all teachers and practitioners of medicine.

The publishers have discharged their duty towards this work with their accustomed fidelity.

These works may be found at the book store of Beckwith & Morton. B.

